# A User-Friendly Software for Automated Knowledge-Based Virtual Surgical Planning in Mandibular Reconstruction

**DOI:** 10.3390/jcm14134508

**Published:** 2025-06-25

**Authors:** Niclas Hagen, Christian Freudlsperger, Reinald Peter Kühle, Frederic Bouffleur, Petra Knaup, Jürgen Hoffmann, Urs Eisenmann

**Affiliations:** 1 Institute of Medical Informatics, Heidelberg University, 69120 Heidelberg, Germany; niclas.hagen@med.uni-heidelberg.de (N.H.);; 2 Department of Oral and Maxillofacial Surgery, Heidelberg University Hospital, 69120 Heidelberg, Germany; 3 Oral & Maxillofacial Surgery Service, Te Toka Tumai Auckland, Te Whatu Ora—Health New Zealand, Auckland 1023, New Zealand

**Keywords:** computer-assisted surgery, virtual surgical planning, mandible, reconstruction, fibula, knowledge-based, artificial intelligence

## Abstract

**Background/Objectives**: Virtual surgical planning (VSP) has become the gold standard in mandibular reconstructions with autografts. While commercial services are available, efforts are under way to address their shortcomings, which may include inefficiency, inconvenience, and susceptibility to error. We developed a novel approach to calculate knowledge-based reconstruction proposals. The objective of our work is to implement software for automated VSP and to evaluate it on retrospective clinical cases. **Methods**: We developed software, which incorporates registration of a naturally shaped mandible, tumor resection planning, knowledge-based calculation of reconstruction proposals, and manual refinement of proposals. Three surgeons planned 21 retrospective clinical cases utilizing our software. They rated its usability via the System Usability Scale (SUS) and rated the quality of the proposed reconstructions and the final surgical plan via a five-point Likert scale (1: totally disagree–5: totally agree). **Results**: Surgeons rated the usability with an average SUS score of 76.7. Times for VSP were consistently less than 20 min. The surgeons agreed with the proposals with a mean value of 4.7 ± 0.4. In 15 cases they made minor refinements. Finally, they agreed with the final surgical plan in twenty cases (score of 5) and with minor discrepancies in one case (score of 4). **Conclusions**: We developed an easy-to-use software for the automated VSP of mandibular reconstructions with autografts. The results demonstrate that reconstruction proposals can be calculated efficiently based on standardized rules. Our system allows surgeons to autonomously derive, compare, and rapidly refine high-quality reconstruction proposals based on key decisions.

## 1. Introduction

Virtual surgical planning (VSP) has become the gold standard for preoperative planning in mandibular reconstruction when using autologous transplants (autografts) [1,2,3,4]. Compared to conventional methods, VSP was associated with improved outcomes, including more precise reconstruction, reduced intraoperative and ischemia periods, reduced hospital stays, fewer complications, and improved quality of life [5,6,7,8]. Typically, the surgeon conducts a virtual meeting with an industry partner to plan the mandibular reconstruction. Usually, an industrial engineer directs the planning and is guided by the surgeon [3,9,10,11]. While associated with better outcomes, this process has shortcomings as it is inefficient, lengthy, cumbersome, and error-prone [7,8,12,13,14,15]. Planning can also complicated by communication barriers [7] and costly, with planning costs ranging from €2500 to €8000 per case [4,16]. While existing commercial services are appreciated, various experts emphasize the need for further scientific developments to provide cost-effective, efficient, precise, and reliable VSP [8,15].

### 1.1. Related Works

Scientific works cover the pre-, intra-, and postoperative treatment phases in reconstructive surgery. Various groups are working on statistical shape models to provide a close approximation of the native morphology as a basis for precise VSP [17,18,19]. Biomechanical models are utilized to address real-world forces in virtually reconstructed mandibles [20,21,22]. Aftabi et al. simulate expected functional outcomes of VSP to support surgical decision-making [23]. Dogan et al. and Li et al. designed patient-specific implants (PSI) using commercial software, and carried out a finite element analysis (FEA) to simulate the force ratios of VSP with custom plates [21,22].

In recent years, significant advances have been made through artificial intelligence (AI) in craniomaxillofacial reconstruction and bone movement. Deep learning (DL) especially enables efficient pre-processing through the automated segmentation of relevant bone structures [24,25,26,27]. Xu et al. have developed algorithms based on generative AI for intelligent VSP of orbital wall reconstructions [28]. Wodzinski et al. report a U-Net-based cranial defect reconstruction and the subsequent calculation of a customized implant [29]. In orthognathic surgery, Kim et al. described a CNN-based approach for automated planning of jaw movements based on 2D cephalograms [30]. Xiao et al. focus on the DL-based prediction of a reference mandible in deformed jaws during orthognathic surgery [31]. A methodologically related emerging trend is VSP for facial feminization surgery [32]. In the context of mandibular reconstruction, Liang et al. describe a generative AI approach to recover the mandibular morphology [33]. Nevertheless, conventional methods continue to be preferred when it comes to reconstruction planning. Besides established CAD/CAM approaches [34], a number of slightly automated in-house procedures with mostly commercial software applications are reported [35,36,37,38]. On the other hand, various groups are working on dedicated software applications for VSP. For example, Maisi et al. utilized the open-source 3D Slicer plug-in *BoneReconstructionPlanner* [39]. These groups are focusing on interactive CAD approaches and semi-automatic planning, with an emphasis on restoring natural mandibular morphology [40,41,42,43].

Despite the wide range of applications, the above-mentioned efforts lack important methodological concepts to achieve automated and qualitatively improved VSP outcomes for mandibular reconstructions with autografts. We believe that semantic networks pave the way for the integration of comprehensive surgical domain knowledge for an in-depth VSP. Such networks could be interpreted by planning algorithms and used for reasoning. This allows for profound functional (e.g., jaw relation) and aesthetic (e.g., morphology or symmetry) considerations during automated surgical planning.

### 1.2. Contributions

In preliminary work, we developed a novel AI approach to calculate knowledge-based reconstruction proposals automatically [44]. To use the proposed algorithms in routine clinical practice, we introduce an easy-to-use software application. The contributions of this work are as follows:

- Description of an automated VSP workflow with standardized planning activities in tumor-related mandibular reconstruction.
- Introduction of an easy-to-use self-developed software that permits surgeons to plan mandibular reconstructions via free fibula flaps swiftly and without relying on external planning services.
- Automated planning assistance for knowledge-based calculation of reconstruction proposals, which can be easily parameterized via the user interface.
- Evaluation of 21 heterogeneous clinical cases to assess the usability and to demonstrate the feasibility of automated planning with high quality using our software.

## 2. Materials and Methods

### 2.1. Preliminary Work

We reported a novel AI approach for knowledge-based mandibular reconstruction in a preliminary article [44]. Our approach is based on an extensive ontology comprising approximately 7500 lines of code that we have developed in-house and which includes standardized rules for reconstruction and a vast collection of functional and aesthetic guidelines for optimized transplant positioning. The ontology contains knowledge on segmental mandibular defects and possible variants for reconstruction with a free fibula flap. The variants differ in their number of segments, segment positioning in the recipient region, donor site used, orientation of additional skin island, as well as the location of the vascular pedicle for anastomosis. Moreover, we developed a generic 3D mesh reference, which defines the target geometry of a natural human mandible. It contains pre-defined anatomical landmarks for an elastic registration of the reference with the patient’s mandible, rigid reference points for transplant positioning, as well as marked bone ridges and the mandibular midline. In an iterative process, our planning algorithm, considering anatomic restraints, dynamically interprets underlying surgical knowledge and calculates an optimized reconstruction proposal for every possible reconstruction variant described in the ontology. The reference model is utilized to automatically apply the interpreted knowledge to the individual anatomy of the patient.

### 2.2. Software for Knowledge-Based Planning

Our self-developed software for VSP of tumor-related mandibular reconstruction is based on the Medical Imaging Interaction Toolkit (MITK) Version 2018.2 (German Cancer Research Center (DKFZ), Heidelberg, Germany) and is implemented in C++. We used Visual Studio 2017 (Microsoft Corporation, Redmond, WA, USA) as the development environment. We implemented the software iteratively in an agile process and with iterative feedback from the oral and maxillofacial surgeons who we continuously consulted to ensure meeting their needs.

As input, the software requires preoperative volume images (usually CT or DVT), as well as segmented bone structures of the mandible, the residual cranium, and the fibula in one or both legs. Segmented peroneal vessels may be considered if Angio-CT is available.

**Figure 1:**
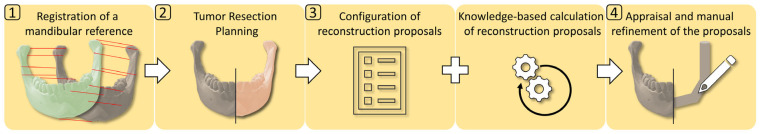
Workflow of a virtual surgical planning process with the developed software.

The software’s workflow is illustrated in Figure 1. It consists of four steps: (1) registration of a mandibular reference, (2) tumor resection planning, (3) configuration and automatic calculation of reconstruction proposals, and (4) appraisal and manual refinement of the proposals. The software guides the user step by step through the planning process. An undo/redo concept allows for movement between completed workflow steps. For guided dialogue, we provided individual graphical elements in the user interface (UI) and the image data required for visualization at each step.

In a four-window view, the head-and-neck region is displayed with 2D projections of sliced images and a 3D view. Additionally, we implemented a separate 3D visualization for the donor site.

#### 2.2.1. Registration of a Mandibular Reference

**Figure 2:**
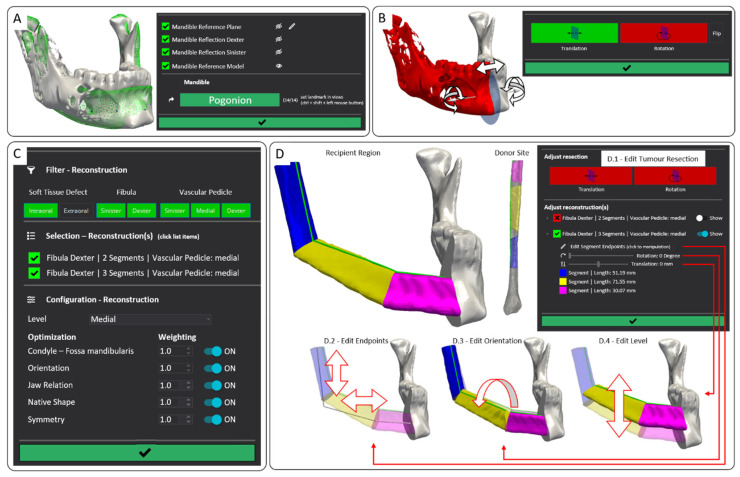
The 3D visualizations and user interfaces (UI) of the different planning software process steps. (**A**): Three-dimensional visualization of the registered mandibular reference (green) and the patient’s mandible (grey). The UI (**on the right**) displays the landmark to be positioned (in this example: Pogonion) on the patient’s mandible. (**B**): Three-dimensional visualization of the planned tumor resection (red) and the residual mandible (grey). The incision is indicated as a blue cut plane. After enabling on the UI (green button on the right), a cut plane can be interactively manipulated (white arrows). (**C**): The UI for the filtering, selecting, and configuring of reconstruction variants for which a proposal shall be calculated by the software. (**D**): Three-dimensional visualization of a proposed reconstruction (residual mandible (grey) and three transplant segments (blue, yellow, and pink)). The software offers the possibility of adapting the tumor resection cut plane (**D.1**), freely editing the segment endpoints (**D.2**), rotating the whole transplant (**D.3**), and manipulating the level (**D.4**). These functions are accessible via the UI for refinement of the transplant.

For the registration process, our mandibular reference provides 14 pre-defined anatomical landmarks evenly distributed throughout the 3D model to capture its characteristic shape accurately. Corresponding landmarks on the patient’s mandible are required to fit the reference. Therefore, the planning software provides a registration wizard, which walks the user step-by-step through the list of landmarks to be placed on the patient’s mandible (Figure 2A). The user can freely move and position landmarks within the 2D slices or on the 3D representation of the segmented mandible. In case of unilateral defects, the healthy side of the mandible can be mirrored at its median plane and used instead of the diseased one. The quasi-symmetrical shape of the mandible facilitates reliable landmark positioning, even in severe deformities. However, if a landmark cannot be placed properly, the user can simply skip it via the UI. Finally, the elastic registration is automatically calculated and visualized for which at least six corresponding landmarks are required [44].

#### 2.2.2. Tumor Resection Planning

When planning the resection of a tumor, the user defines the part of the mandible to be removed by placing one or two planar cuts (depending on the case) on the 2D slices of the volumetric image or on the 3D representation of the segmented mandible (Figure 2B). To ensure the cuts are located on the mandible (the region of interest), they are projected automatically onto the nearest point of the registered mandibular midline. Furthermore, the cuts are aligned perpendicular to the bone surface to prevent any inappropriate edges. A grab-and-drag mechanism can be activated to move the cut planes along the mandibular midline or to rotate them around their center for further adjustment (Figure 2B). The bone planned to be removed is highlighted in red in real-time for an intuitive visualization.

#### 2.2.3. Configuration and Automatic Calculation of Reconstruction Proposals

All available transplant variants for bridging the planned resection are retrieved from the ontology, which has been previously described [44]. The variants are presented to the user with information on the number of bone segments, the donor site from which they are harvested, and the location of the vascular pedicle for anastomosis in the recipient region (Figure 2C). Filtering options can narrow down the search results based on the donor bone, recipient region anastomosis, or orientation of an additional skin island. The user can interactively select all the variants for which the planning software will automatically calculate a reconstruction proposal.

In addition, the user can configure the algorithm for calculating knowledge-based reconstruction proposals to suit individual needs (Figure 2C). Thus, the software offers options for setting the level (height) of the transplant in the tooth-bearing part of the mandibular body, including basal, middle, and apical heights. It is also possible to enable or disable various functional and aesthetic analyses and define their weighting to optimize the transplant. The default configuration is balanced, meaning all aspects are given equal attention.

After software configuration, the proposals are iteratively calculated without further input by the user [44]. The software automatically considers important parameters such as correct segment positioning, segment lengths, precisely fitting cut edges, and the lateral segment surface forming the lateral surface of the reconstructed mandible, as well as medially running vessels. Thus, patient-specific reconstruction proposals are calculated based upon the ontology’s knowledge and according to standardized rules.

#### 2.2.4. Appraisal and Manual Refinement of the Proposals

Finally, the resulting proposals can be viewed, compared, and fine-tuned manually (Figure 2D). The software offers various concepts for direct manipulation of the transplant in real time:

- The segment edges can be interactively manipulated in the 2D and 3D views of the head-and-neck region. At the junction with the residual mandible, only a displacement on the planned cut edge is permitted to ensure a seamless connection.
- To adjust the transplant height, the segments in the tooth-bearing part of the mandibular body can be moved by a slider on the UI.
- The transplant can be rotated around the mid-axis via a slider to adjust its lateral surface.
- As with tumor resection planning, the cut planes can be re-positioned and re-oriented afterwards.

The software provides a preview of all modifications and updates the reconstruction proposals automatically. It also alerts the user when segment lengths are shorter than allowed parameters (≤2.5 mm). Ultimately, a single reconstruction must be chosen as the final planning result.

### 2.3. Data Selection

To evaluate our software, we selected 21 retrospective, primary mandibular reconstructions related to tumors from the Department of Oral and Maxillofacial Surgery in Heidelberg. The study cases were chosen to represent a typical distribution of different severities and HCL classes according to Jewer et al. [45] (Table 1). For each case, we exported the preoperative volume images of the head-and-neck and lower extremities from the Picture Archiving and Communication System (PACS) and anonymized the data. The evaluation was approved by the local ethics committee.

### 2.4. Pre-Processing

An experienced medical informatician segmented the relevant bone structures for VSP, which included the mandible, residual cranium, and fibulae utilizing the MITK Workbench [46]. The segmentation masks were accurately defined through a 3D region growing and a subsequent manual refinement. We triangulated the 3D surface representations via the MITK Workbench. The peroneal arteries were determined with the MITK Workbench when Angio-CT was available. The vessels were marked by manually placing landmarks on the 2D slices of the leg images. A 3D representation of the vessels was calculated by approximating the landmarks with a Kochanek–Bartels spline [47].

### 2.5. Virtual Surgical Planning of Retrospective Clinical Cases

The planning software accurately runs on a notebook with an Intel Core i7-8665U (1.90 GHz) processor (Intel Corporation, Santa Clara, CA, USA), 16 GB of RAM, and an NVIDIA GeForce MX250 graphics card with 2 GB of dedicated GPU memory (NVIDIA Corporation, Santa Clara, CA, USA). It can be used anywhere in the hospital without requiring an internet connection or external computing power.

A medical informatician registered the mandibular reference for each case and saved it as an intermediate result. Tumor resection and mandible reconstruction were then planned by three surgeons after loading the intermediate result for the registered reference. The 21 study cases were divided equally among the surgeons based on the HCL classification, with seven virtual surgical plans for each surgeon. Prior to the actual planning of the assigned cases, the surgeons familiarized themselves with the software by training with a non-study case.

After planning a study case, the surgeons evaluated its complexity, the proposed reconstructions, and the final surgical plan on a five-point Likert scale (1 = Strongly disagree; 5 = Strongly agree) by answering the following questions:

- Complexity: I consider the complexity of planning this case to be high.
- Proposal: I consider the proposed reconstruction to be suitable.
- Final Surgical Plan: I consider that the mandibular reconstruction can be planned as desired (if necessary, after manual refinement of the proposal).

To assess the software’s usability, the surgeons completed the validated System Usability Scale (SUS) questionnaire [48]. The software automatically stored detailed time logs during VSP.

## 3. Results

The surgeons assessed the usability of the developed planning software with an average SUS of 76.7 (surgeon 1: 77.5; surgeon 2: 75.0; surgeon 3: 77.5). A SUS scale > 68 is considered above average and corresponds to a grade of *B* or a *good* usability.

Table 2 shows the measured times for VSP for each of the 21 study cases. The average time for registration of the mandible reference was 5.4 ± 1.4 min. The surgeons spent on average 12.0 ± 5.3 min on planning the tumor resection and the mandible reconstruction. Thereby, planning a tumor resection took 3.2 ± 1.6 min, while the configuration of the reconstruction proposals took approximately 1 min. Finally, appraisal and manual refinement of the proposals was completed in 5.4 ± 2.4 min. We observed noticeable differences during the planning of the resection between surgeon 2 (requiring less than 2 min) and surgeon 3 (requiring on average 5.0 ± 1.4 min).

The automatic calculation of the reconstruction proposals took on average 15.0 ± 0.1 s. The shortest time of 3 s was recorded for a case where only one proposal, consisting of two segments, was calculated. The maximum duration of 50 s was recorded for a case where four proposals were calculated including two proposals with 3 segments each and two proposals with 4 segments each.

The surgeons classified the study case complexities as moderate to high, with a complexity score ranging from 1 (not complex) to 5 (highly complex). Some correlation was found between the estimated complexity and the number of transplant segments used for reconstruction (1 segment: 2.2 ± 0.5; 2 segments: 3.5 ± 0.8; 3 segments: 4.8 ± 0.4; 4 segments: 5). Moreover, there were differences in complexity perception between the surgeons (surgeon 1: 4 ± 1; surgeon 2: 4.5 ± 0.7; surgeon 3: 2.5 ± 0.5).

**Figure 3:**
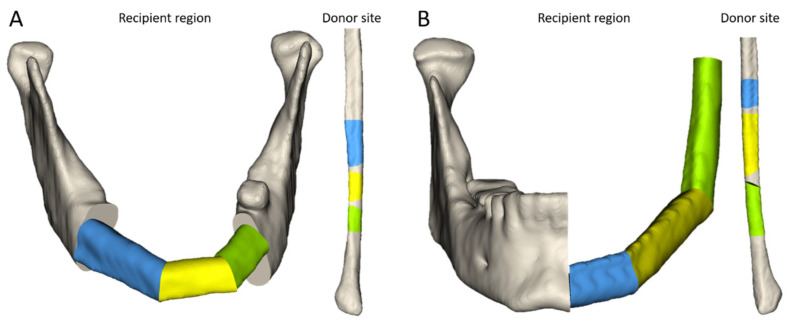
Successfully calculated reconstruction proposals. (**A**): Recipient region and donor site of a class LCL reconstruction with three fibula segments (green, yellow, blue). (**B**): Recipient region and donor site of a class H reconstruction with three fibula segments (green, yellow, blue).

The reconstruction proposals were calculated for all 21 study cases successfully (Figure 3A,B). The surgeons agreed with the proposals with a mean value of 4.7 ± 0.4 (1 (totally disagree)–5 (totally agree)). In fifteen study cases, the automatically calculated proposals were rated with a score of 5, while in six cases, they were rated with a score of 4. However, the surgeons made manual adjustments in 15 cases. The most pertinent adjustments and the surgeons’ feedback during refinement of the proposals are described in Table 3.

**Figure 4:**
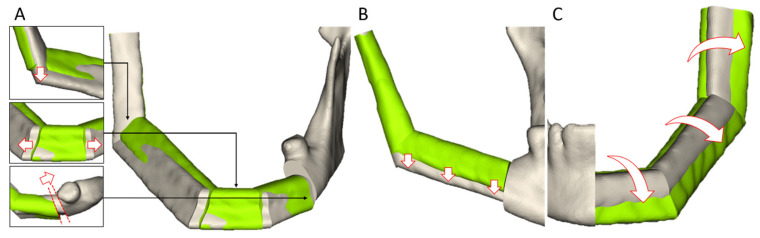
Comparison of proposed reconstructions (green) and final plan (grey) after manual refinement for three study cases. (**A**): In a large mandibular defect with four segments, a manual correction of the reconstructed jaw angle towards the mandibular base was performed, as well as a refinement of the anterior segment, which was found to be too short. Furthermore, an adjustment of the tumor resection was carried out. (**B**): A reassessment of the previously defined transplant level was conducted for a Class H reconstruction after viewing the proposed reconstruction, resulting in a subsequent manual adjustment of the transplant level. (**C**): A re-orientation of the proposed transplant was performed to ensure the most natural bone surface possible.

While viewing the proposed reconstructions, a total of six study cases were subjected to a reassessment of the previously defined configuration of the calculation algorithms. Thereby, adjustments were made to the tumor resection (*n* = 3) and the manually selected transplant level (*n* = 3) (Figure 4A,B). Most of the observed refinements pertained to the correction of the proposed reconstruction in the jaw angle (*n* = 5), and to the orientation of the transplant (*n* = 4) (Figure 4A,C). In two cases, the surgeons adjusted segments which they deemed too short (Figure 4A). In two further cases, a standard mono-segment reconstruction variant for short mandibular defects was suggested.

Following the manual refinement, the surgeons perfectly agreed (score of 5) with the final planning result in 20 cases. Minor discrepancies (score of 4) were observed in one wide-spanned transplant with four segments (Figure 4A).

## 4. Discussion

We presented in this manuscript our user-friendly software for knowledge-based virtual surgical planning of mandibular reconstructions. The evaluation on retrospective clinical cases demonstrated promising results regarding an efficient in-house approach at the point of care. It empowers surgeons to plan independently without industry consultation and with high quality, even in the event of an immediate intervention being necessary.

The software is characterized by a high level of automation, reducing the number of complex considerations while ensuring compliance with essential reconstruction rules. This facilitates the standardization of transplant planning, enabling surgeons to examine a reconstruction in greater detail. Concurrently, the software provides a variety of functions for surgeon-specific planning (e.g., manual tumor resection planning) and manual refinement of proposed transplants.

The software was developed in an agile process in close collaboration with surgeons, allowing for the continuous incorporation of feedback from prospective users. Consequently, its functionalities and UI are strongly oriented towards the surgeon’ needs, as evidenced by the *good* SUS (76.7) obtained during evaluation. The MITK was selected as a modular development framework with a broad range of reusable functionalities (e.g., visualization concepts, interaction concepts, and registration algorithms) for computer-assisted surgery [49]. A modular structure is also evident in the software’s workflow, comprising self-contained steps, which guide the user through a planning session. This facilitates the maintenance and extension of our software. Furthermore, this enables the portioning of planning tasks into discrete stages, which can be conducted by a technical assistant (registration mandibular reference), and the surgeons (resection and reconstruction planning).

The evaluation demonstrates that the virtual surgical planning of a mandibular reconstruction can be accomplished in less than 20 min utilizing our software. This is applicable to single-segment and more complex multi-segment transplants. Discrepancies between surgeons during VSP are attributable to individual technical skills and the relatively short training phase on one single non-study case. These findings demonstrate the necessity of comprehensive training for end users, even when utilizing an easy-to-use software with the capacity to automatically generate planning proposals.

The evaluation of the proposed reconstructions on clinical cases revealed no significant differences between the surgeons or between the different HCL classes of the study cases. With an average clinical rating of 4.7 ± 0.4, the proposals were identified to be of high quality. The findings demonstrate that the complexity of VSP for mandibular reconstructions has been successfully reduced to a few central configurations, including the donor site to be used, or the level of the transplant, which must be defined by the surgeon. Moreover, all variants to bridge a mandibular defect are identified automatically on the basis of the underlying knowledge. We have achieved a standardization of high-level VSP in mandibular reconstruction while still allowing surgeon-specific planning by simply parametrizing a few settings. Thus, surgeons only have to make some key decisions to tailor the transplant to their specific needs.

Approximately one-third of the proposed reconstructions were accepted without further modifications, while the surgeons made minor refinements to the remaining 15 study cases. In six cases, the appraisal of the 3D-visualized proposals led to a reassessment of previously defined tumor resection (*n* = 3) and transplant level (*n* = 3). Here, the proposed reconstructions can be considered a high-quality starting point for a more in-depth VSP. This exemplifies the iterative process generally inherent to virtual reconstruction, which can also be observed in clinical practice during web-based VSP. In this context, a human-in-the-loop approach could improve our automated calculation of reconstruction proposals and allow the user to intervene at this point. Such approaches are well-known in the field of machine learning [50]. When transferred to our topic, intermediate reconstruction results could be presented to the user in real time, to incorporate surgeon-specific preferences into the calculation process. This may also reduce the subsequent refinement effort.

The surgeons’ feedback during the evaluation indicated potential for improvements in the positioning of the transplant parallel to the occlusal plane, achieving a more balanced orientation to account for a naturally shaped neo-mandible, and the prevention of too-short segments. At present, our software is capable of reliably identifying short segments and alerting the user. Nevertheless, potential necrosis of short segments [51] should be considered automatically by our algorithms in the future.

In the event of short mandibular defects, a mono-segment solution could be simply added as a standard variant, as suggested by the surgeons. Due to the separate knowledge management implemented in our software, these variants could be incorporated into the ontology without any adjustments to the algorithms.

Finally, all 15 manually refined cases could be adjusted by the surgeons in a short time (on average 5.4 ± 2.4 min). This indicates that, even in severe cases, the proposed reconstructions offer at least a highly viable basis for refinement. The functionalities provided represent efficacious and effective concepts for the precise manipulation of a transplant.

Similar systems for creating and subsequently refining digital treatment plans can be found in orthodontics to correct malocclusions. In this field, Invisalign (Align Technology, San Jose, CA, USA) is a leading provider of clear aligner treatment. With their application, it is possible to plan optimized tooth positions and simulate the intermediate stages in which the teeth move into their optimal position [52]. However, these systems do not provide rules tailored to our purpose that can be used to automate the calculation of an optimized mandibular reconstruction via autografts.

Our software has been implemented with a user-friendly UI and interaction concept, accompanied by a dedicated visualization. A similar application, emphasizing usability and comprehensive planning tasks, is reported by Olsson et al. [53]. They are focusing on a haptic interaction, offering the user a high degree of freedom. Nevertheless, their application lacks a methodology for automated positioning of the transplant, which would greatly assist the surgeon in achieving optimal planning results. Furthermore, the reported durations for planning three cases ranged from 29 to 63 min, which is considerably longer than the times (less than 20 min) we observed in 21 cases with our software.

Compared to approaches which report on automation of reconstruction [40,41,42], we consider various functional and aesthetic aspects during the calculation of knowledge-based reconstruction. This enables more profound proposals. Moreover, most works focus on reconstructing the natural shape or the symmetry of the patient’s mandible by determining a reference line (e.g., the midline or a baseline of the mandible), to which the transplant is matched. Nakao et al. approximate the patients’ mandible shape directly [41,54]. In contrast, we consider an anatomically correct standard model of the mandible as a reference, rather than potentially deformed parts of the patient’s mandible. This is a crucial advantage for the reliability and quality of VSP. Furthermore, our software provides a list of variants for bridging a mandibular defect. Finally, the proposals can be compared and individually refined with the provided manipulation concepts to find the optimal solution.

In contrast to web-based planning with an industrial provider [3,8,9,11], our software enables more efficient process cycles by incorporating a broad range of knowledge to support the surgeons as effectively as possible. This eliminates the need to reiterate the knowledge at each planning meeting. Surgeons are able to directly parameterize and manipulate the surgical plan with our software. These are crucial steps to an efficient patient- and surgeon-specific VSP of mandibular reconstructions at the point of care. Subsequently, the results can be utilized to generate cutting guides and to manufacture or pre-bend patient-individual reconstruction plates in the clinic [55,56,57].

Nevertheless, our work has some limitations. The measured planning periods did not include time-consuming pre-processing steps, such as image acquisition. One of the most significant constraints is the lack of an automated segmentation in our software. In recent years, deep learning approaches have been described with promising results in automated segmentation [24,26,27,58]. Another limitation concerns the sole support of fibula transplants. Thus, it is necessary to evaluate the applicability of our software to other autografts like the iliac crest or scapula. Further potential can be seen in the registration of the mandibular reference. A substantial amount of time (approximately 5.4 ± 1.4 min) was required to position anatomical landmarks for registration. However, various AI-based approaches for automated landmark detection have been described [59,60]. Alternatively, shape models and biomechanical models could be utilized as a reference to consider the statistical variances of the mandible [12,17,18,19,20]. Based on this, gender-differentiated models could be employed to achieve even more precise and natural results.

We see further potential in comprehensive backward planning [3,10]. The simultaneous planning of implants for dental rehabilitation [57] would be a decisive advantage to our approach. In addition to the aforementioned limitations, a larger clinical study in a multi-center setting is desirable. This would allow for an in-depth analysis of our software, which operates on the knowledge acquired with the oral and maxillofacial surgeons from our clinic. At present, the evaluation by surgeons from one’s own clinic may be considered as a possible bias in the assessment of the proposed reconstructions.

## 5. Conclusions

We present an easy-to-use software for automated VSP of mandibular reconstructions with autografts, based on a novel knowledge-based approach. The results indicate that our software can be utilized to calculate reconstruction proposals automatically applying standardized rules. The proposals can be easily and efficiently refined by the user. Thus, our approach facilitates precise, efficient, and reliable VSP at the point of care. Subsequently, the planning outcomes can be utilized for the preparation of patient-specific plates and cutting guides to transfer the surgical plan into the operating theatre. In summary, our system allows surgeons, for the first time, to autonomously derive, compare, and rapidly refine high-quality reconstruction proposals based on key decisions.

## Figures and Tables

**Table 1:** Number of study cases sorted by HCL class and location of the defect in the recipient region (central, left, right).

HCL Class	Centered Defects	Left-Sided Defect	Right-Sided Defect	Overall
**H**	-	6	4	10
**HCL**	-	0	1	1
**L**	-	2	2	4
**LC**	-	2	2	4
**LCL**	2	-	-	2
**Overall**	2	10	9	21

**Table 2:** Measured times (in minutes) for virtual surgical planning of all study cases via the developed software.

Planning Step	Medical Informatician	Surgeon 1	Surgeon 2	Surgeon 3	Overall
**Registration mandibular reference**	5.4 ± 1.4	-	-	-	-
**Load and view registration**	-	1.3 ± 0.4	1.2 ± 0.5	2.4 ± 1.4	1.5 ± 1.2
**Tumor resection planning**	-	2.4 ± 1.6	1.6 ± 0.3	5.0 ± 1.4	3.2 ± 1.6
**Configuration**	-	0.5 ± 0.4	0.5 ± 0.3	1.4 ± 2.1	1.1 ± 1.2
**Calculation of reconstruction proposals**	-	0.2 ± 0.2	0.2 ± 0.1	0.1 ± 0.0	0.2 ± 0.1
**Appraisal and refinement of the proposals**	-	5.3 ± 3.4	4.5 ± 1.6	6.3 ± 2.1	5.4 ± 2.4
**Overall**	-	10.4 ± 5.2	9.2 ± 2.4	16.1 ± 6.1	12.0 ± 5.3

**Table 3:** Manual refinement and the surgeons’ feedback on the proposed reconstructions of the software during virtual surgical planning of the study cases. The aspects are categorized, with *n* indicating the number of mentions. A study case may comprise several aspects.

**Manual Refinement/Adaption of …**	**Description/Feedback of the Surgeons**
**Tumor Resection** (*n* = 3)	Reassessment of the tumor resection after appraisal of the reconstruction proposals.
**Transplant Level** (*n* = 3)	Reassessment of the configured transplant level after appraisal of the reconstruction proposals.
**Reconstructed Jaw Angle** (*n* = 5)	Regarding the postoperative restoration with dental implants, it is necessary to correct the proposed transplant in the jaw angle (towards the mandible base) to ensure parallel positioning to the occlusal plane.
**Short Segments** (*n* = 2)	Correction of short transplant segments in the anterior region of the mandibular body to prevent necrosis.
**Jaw Relation** (*n* = 1)	Correction of the proposed graft in the anterior part of the mandible, which exhibits an asymmetrical alignment with the maxilla.
**Reconstructed Jaw Joint** (*n* = 1)	To prevent the jaw joint and the fossa mandibularis from tilting, it is necessary to refine the proposed transplant due to an overlap with the skull bone.
**Orientation** (*n* = 4)	Refinement of the orientation (rotation) for a smooth transition between the transplant and the residual mandible, or to ensure a natural bone surface.
**Mono segment reconstruction** (*n* = 2)	For short mandibular defects, a mono-segment reconstruction variant should be offered by default, even if the defect extends over prominent structures like the jaw angle.

## Data Availability

The datasets presented in this article are not readily available because of legal restrictions. Requests to access the datasets should be directed to the corresponding author.

## Abbreviations

The following abbreviations are used in this manuscript:

VSP	virtual surgical planning
PSI	patient-specific implants
FEA	finite element analysis
AI	artificial intelligence
DL	deep learning
CNN	convolutional neural network
CAD	computer-aided design
CAM	computer-aided manufacturing
MITK	Medical Imaging Interaction Toolkit
CT	computer tomography
DVT	digital volume tomography
UI	user interface
PACS	Picture Archiving and Communication System
RAM	Random-Access Memory
GB	Gigabyte
GPU	graphics processing unit
SUS	System Usability Scale
